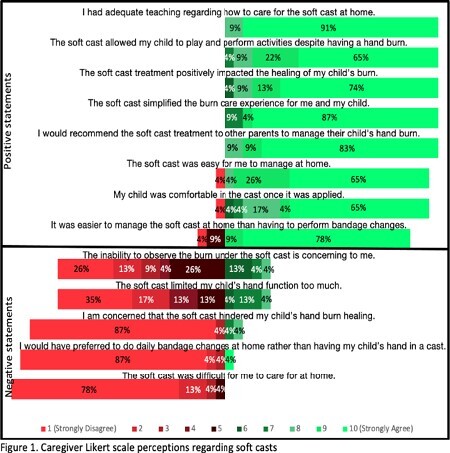# 562 Caregivers and Clinic Providers View Soft Casts for Pediatric Hand Burns as Acceptable and Feasible

**DOI:** 10.1093/jbcr/irae036.196

**Published:** 2024-04-17

**Authors:** Jennifer M Schuh, Leanna L Linzell, Emmanuel L Abebrese, Katherine T Flynn-O'Brien

**Affiliations:** Children's Wisconsin, Milwaukee, WI; Children's Hospital of Wisconsin, Wauwatosa, WI; Medical College of Wisconsin, Milwaukee, WI; Children's Wisconsin, Milwaukee, WI; Children's Hospital of Wisconsin, Wauwatosa, WI; Medical College of Wisconsin, Milwaukee, WI; Children's Wisconsin, Milwaukee, WI; Children's Hospital of Wisconsin, Wauwatosa, WI; Medical College of Wisconsin, Milwaukee, WI; Children's Wisconsin, Milwaukee, WI; Children's Hospital of Wisconsin, Wauwatosa, WI; Medical College of Wisconsin, Milwaukee, WI

## Abstract

**Introduction:**

Soft casts have been introduced as a strategy to manage hand burns that is associated with less wound care burden for families. We hypothesized that the use of soft casts in pediatric hand burns would be viewed as acceptable by patient caregivers and providers, perceived as logistically feasible, and result in satisfactory clinical outcomes.

**Methods:**

A retrospective chart reviewed was perform for pediatric patients with hand burns who were managed with soft casts in clinic since implementation in 2022. A survey was sent to patient caregivers (n=33), and a separate survey sent to burn clinic staff and providers (n=20). The primary outcome was perception of acceptability of soft casts as a management strategy (questions targeted care burden, overall satisfaction, ease of use, healing concerns, efficacy, and comfortability). The secondary outcome was provider perception of feasibility of this new strategy (effect on clinic workflow, efficiency). Survey questions were both Likert scale and open ended. Findings were summarized and qualitatively assessed.

**Results:**

Since implementation of soft casting in 2022, 33 English-speaking patients with hand burns were managed with soft casts and 38 soft casts applied. Total body surface area burned ranged from < 0.5% to 2.5%, with both superficial and deep burns treated. There was a mean of 1.8 reapplications (mode 1, range 1-22) and a median time to healing of 13 days. There were no infections attributed to the use of soft casting, and only one patient initially in a soft cast required grafting. Survey responses were collected from 70% of caregivers (23/33) and 90% of providers (18/20). Caregivers overwhelmingly agreed with statements favoring soft cast acceptability and disagreed with negative statements regarding soft casts (Figure 1). Among clinic providers, 78% strongly agreed (9 or 10 on Likert) with the statement “The soft casting method has streamlined our clinic’s operational efficiency for managing hand burns” and 94% would recommend soft casts (range 7-10, mode 10 on Likert). Qualitative thematic analysis of open-ended questions was conducted (Table 1).

**Conclusions:**

Overall, the introduction of soft casts as a management strategy for pediatric hand burns was well received by patient caregivers and clinic team, and felt to be acceptable (satisfactory, comfortable, practical, and efficacious) and feasible, with little disruption to clinic flow. The clinical outcomes assessed suggest soft casts are associated with good wound healing with minimal wound care for patient and family.

**Applicability of Research to Practice:**

The perceived acceptability of soft casts for pediatric hand burn treatment and feasibility of integrating this as a new management strategy into a busy clinic speak to the pragmatism of logistically integrating soft casts into practice and support the strategy as satisfactory to parents and providers.